# The Art of Interpreting Antinuclear Antibodies (ANAs) in Everyday Practice

**DOI:** 10.3390/jcm14155322

**Published:** 2025-07-28

**Authors:** Marcelina Kądziela, Aleksandra Fijałkowska, Marzena Kraska-Gacka, Anna Woźniacka

**Affiliations:** Department of Dermatology and Venereology, Medical University of Lodz, pl. Hallera 1, 90-647 Lodz, Poland; marcelina.kadziela@stud.umed.lodz.pl (M.K.); aleksandra.fijalkowska@stud.umed.lodz.pl (A.F.); marzena.kraska@umed.lodz.pl (M.K.-G.)

**Keywords:** ANA, antinuclear antibodies, SLE, systemic lupus erythematosus, mixed connective tissue diseases

## Abstract

Background: Antinuclear antibodies (ANAs) serve as crucial biomarkers for diagnosing systemic autoimmune diseases; however, their interpretation can be complex and may not always correlate with clinical symptoms. Methods: A comprehensive narrative review was conducted to evaluate the peer-reviewed literature published between 1961 and 2025. Databases, including PubMed and Scopus, were searched using combinations of controlled vocabulary and free-text terms relating to antinuclear antibodies and their clinical significance. The objective was to gather and synthesize information regarding the diagnostic utility and interpretation of ANA testing in routine medical practice. Discussion: The indirect immunofluorescence assay (IIF) on HEp-2 cells is established as the gold standard for detecting ANAs, facilitating the classification of various fluorescent patterns. While a positive ANA test can suggest autoimmune disorders, the presence and titre must be interpreted alongside clinical findings, as low titres often lack diagnostic significance. Findings indicate that titres higher than 1:160 may provide greater specificity in differentiating true positives from false positives in healthy individuals. The study also emphasizes the relevance of fluorescence patterns, with specific patterns linked to particular diseases, although many do not have strong clinical correlations. Moreover, certain autoantibodies demonstrate high specificity for diseases like systemic lupus erythematosus (SLE) and mixed connective tissue disease (MCTD). Ultimately, while ANA testing is invaluable for diagnosing connective tissue diseases, healthcare providers must consider its limitations to avoid misdiagnosis and unnecessary treatment. Conclusions: ANA testing is a valuable tool in the diagnosis of connective tissue diseases, but its interpretation must be approached with caution. Clinical context remains crucial when evaluating ANA results to avoid misdiagnosis and overtreatment. This review is about the diagnostic aspects and clinical consequences of ANA testing, as well as highlighting both the diagnostic benefits and the potential limitations of this procedure in everyday clinical practice. The review fills a gap in the literature by integrating the diagnostic and clinical aspects of ANA testing, with a focus on real-world interpretation challenges.

## 1. Introduction

In physiological conditions, antibodies bind to specific antigens, allowing the neutralization and elimination of pathogens such as bacteria or viruses. However, in the case of autoimmune diseases, antinuclear autoantibodies (ANAs) are directed against host cells. These antibodies of the IgG isotype target solid or soluble antigens located in the cell nucleus or cytoplasm. Thus, they serve as serological biomarkers in the diagnosis and classification of various systemic autoimmune diseases.

However, while determining the presence and specificity of ANAs may play an important part in management, a positive result does not always confirm a diagnosis or correlate with disease severity. The presence, titre and specificity of ANAs should always be interpreted in relation to other laboratory test results and the patient’s clinical symptoms. Isolated occurrence of ANAs, i.e., without any complaints or symptoms, may not have clinical implications and may just require monitoring. Even so, a negative result can help rule out certain autoimmune diseases [1].

It is also important to remember that, in addition to autoimmune conditions, ANAs may be present in early-stage cancer or in pre-cancerous conditions. Specific ANAs have been detected in patients with lung cancer, lymphoma, thymoma, colorectal cancer, prostate cancer, breast cancer or squamous cell carcinoma, among others [2]. Also, patients with infectious diseases can produce ANAs. However, it is problematic to determine whether ANAs are a cause or an effect of infection [3]. Typically, low ANA titres are detected in patients with latent chronic bacterial or viral infections, including tuberculosis, viral hepatitis, syphilis and parasitic diseases [4].

This paper presents the possibilities and limitations related to the interpretation of serological tests regarding ANAs. It fills a gap in the literature by bridging laboratory immunology with clinical decision-making and highlighting the pitfalls of misinterpretation, which often occurs in everyday practice. Thus, this paper is particularly valuable for clinicians, laboratory workers and medical trainees managing autoimmune diseases on a daily basis.

## 2. Gold Standard and Methodological Considerations

The gold standard in the diagnosis of ANAs, and the most frequently used screening method, is the indirect immunofluorescence assay (IIF) performed on epithelial laryngeal cancer cells (HEp-2 and, less often, HEp-2000) [2]. HEp-2 cells are preferred due to the presence of a wide spectrum of antigens located in the large nuclei and cytoplasm. The use of HEp-2 cells as a substrate has led to greater recognition that, in addition to nuclear patterns, cytoplasmic and mitotic cell patterns can also be identified. HEp-2000 is a modified substrate designed to improve sensitivity to certain autoantibodies like anti-Ro/SS-A, while overall detection profiles for other ANA specificities remain comparable between the two.

In the IIF test, autoantibodies from patient serum are bound to a target antigen localized in HEp-2 cells. The resulting complex is recognized and visualized by fluorochrome-linked secondary antibodies specific to the Fc portion of human immunoglobulin G. This test can not only confirm the presence of ANAs but also their titre if the serum is incubated with different dilutions, typically 1:80, 1:160, 1:320, 1:640, etc. The lowest dilution that still produces fluorescence of any pattern is recorded as the ANA titre. IIF testing can also identify the antigen location in the cell based on the fluorescence pattern [3].

This method can simultaneously identify a number of different antibodies and is widely recommended as a first-choice test. However, the method is labour intensive, time-consuming, requires an experienced reader and does not always allow precise antigen identification based on the fluorescence pattern. In addition, inter-observer variability may lead to slight differences in reported titres, which typically varies within one dilution among trained readers, and inter-assay variability may complicate the interpretation of titres between different labs. While control samples with distinct fluorescence patterns and the routine use of positive and negative controls has been introduced to increase precision, the variability between tests means a one-dilution difference is not clinically meaningful. Since the IIF test involves multiple steps and its interpretation is highly subjective, it has certain limitations. A new standardized and objective diagnostic method has been introduced.

If a patient has a positive IIF test for ANAs, the healthcare provider may order additional tests to identify specific types of autoantibodies, depending on the patient’s symptoms or findings on physical examination. Addressable Laser Bead Immunoassay (ALBIA), enzyme-linked immunosorbent assay (ELISA), Fluorescent Enzyme Immunoassay (FEIA) and Chemiluminescent Immunoassay (CLIA) are widely employed for the detection of specific antibodies. The choice of assay depends on various factors, including available resources, required sensitivity and clinical context. These tests typically include 16 soluble antigens, such as nRNP/Sm, Sm, Ro (SS-A) and La (SS-B), among others.

### 2.1. Clinical Significance of Specific Autoantibodies

The serum dilution that could be considered clinically important remains a topic of debate. Hence, various studies have compared the results of ANA tests between various autoimmune diseases and healthy controls. The findings indicate that positive results of tests performed using the IIF-HEp-2 method, i.e., with serum dilutions lower than 1:160, have little clinical value in the diagnosis of CTD [5]. Approximately 10–12% of healthy people aged between 20 and 60 years possess ANAs at a titre of 1:80, 5% at 1:160 and only 3% at 1:320 [6].

When testing for autoimmune diseases, the ANA test has been found to be highly sensitive at lower dilutions (1:40 or 1:80) but can result in many false-positive outcomes. The specificity can be increased by increasing the dilution (1:160). As such, dilutions of 1:160 and higher have been suggested as a helpful threshold in distinguishing potentially true-positive ANA results from the false-positive results observed in healthy individuals [5,7].

In recent years, this 1:160 cut-off has undergone some modification for the diagnosis of systemic lupus erythematosus (SLE). The new EULAR/ACR classification criteria for SLE proposes the presence of ANAs at a titre of 1:80 as the basic condition necessary for diagnosis [8]. A study of approximately 13,000 people with SLE found an ANA titre of 1:80 to have a sensitivity of 98% for detecting SLE, sufficient to be considered an entry criterion for diagnosis [9,10]. At the above dilution, positive results are observed in up to 13.3% of healthy individuals. In such cases, where a low ANA titre is observed in HEp-2 cells, clinicians should prioritize the assessment of clinical manifestations to guide the diagnostic process. This approach helps avoid unnecessary treatments and minimizes the risk of misclassifying patients [11].

### 2.2. Repeat Testing of Antinuclear Antibodies

ANA testing is primarily used for diagnostic purposes and has limited utility in monitoring disease activity or progression. However, there are clinical situations where repeating ANA testing may be indicated, such as in the case of clinical worsening consistent with connective tissue diseases (CTDs) or when evaluating for the emergence of new ANA specificities associated with new or evolving symptoms. Reassessment in these contexts can aid in the diagnostic process and guide further evaluation. Lake and colleagues performed a study in Ontario and indicated that potentially redundant testing was testing among patients with suspected or confirmed connective tissue disease [12]. The latest research also shows that average titres tend to be higher in early disease and decrease over time [13]. The results of repeated ANA tests do not change much over time; therefore, doctors should take into account the necessity of intervention [14].

### 2.3. Classification of ANA Fluorescence Patterns

ICAP (the International Consensus on ANA Patterns) has categorized 30 different HEp-2 immunofluorescence patterns into four main groups: negative (without any fluorescence), nuclear (15 different patterns), cytoplasmic (9 different patterns) and mitotic (5 different patterns) [12].

### 2.4. Main Nuclear Patterns

Several characteristic nuclear fluorescence patterns are commonly identified, including homogeneous, dense fine speckled, centromere, speckled, discrete nuclear dots, nucleolar, nucleolar envelope and pleomorphic patterns [12] (Table 1).

1. The homogeneous pattern is more closely linked to connective tissue diseases (CTDs) than the speckled pattern. It is frequently associated with SLE or drug-induced lupus erythematosus (DILE). In antigen-specific tests, this type of fluorescence often correlates with the presence of anti-dsDNA [13,14].
2. The nuclear dense fine speckled pattern is frequently observed in healthy individuals who possess DFS-70 autoantibodies. This pattern, characterized by speckles of varying sizes, intensities and distributions, is scattered across the interphase nucleus [14].
3. The centromere pattern is a kind of specific speckled staining that is usually connected with the presence of antibodies against centromeres. These antibodies are strongly associated with CREST syndrome, a subset of scleroderma with a generally favourable prognosis. The key features of CREST syndrome include calcinosis (deposition of calcium in soft tissues), Raynaud’s phenomenon (increased sensitivity of fingers and toes to cold), oesophageal dysfunction (often leading to difficulty swallowing), sclerodactyly (skin thickening on the fingers) and telangiectasia (dilated capillaries visible on the skin). Such antibodies are rare in systemic scleroderma and are infrequently detected in other connective tissue disorders [14].
4. Nucleolar fluorescence is characterized by large coarse speckled staining within the nucleus, less than six in number per cell. The nucleolus is a spherical structure found in the nucleus, whose primary function is to produce and assemble ribosomes. This pattern is more common for patients with scleroderma, less frequent in Sjögren’s syndrome or mixed connective tissue disease and is sometimes noted in healthy people with ANAs. Nucleolar autoantibodies react with PM-Scl, RNA polymerase I, NOR-90, RNase P, nucleolin, URNP, U3-RNP, To/Th and B23 phosphoprotein/numatrin [15].
5. The nuclear large/coarse speckled pattern is characterized by the presence of large or coarse speckles distributed across the nucleoplasm. Staining of the nucleoli may or may not occur in this pattern. Examples include antibodies such as anti-Sm and anti-U1 RNP, which are indicative of mixed connective tissue disease (MCTD) [15].

### 2.5. Among the Cytoplasmic Character of Fluorescence, Filamentous, Speckled, AMA, Golgi and Rods and Rings Are Observed Most Often

1. Filamentous fluorescence staining is used to highlight microtubules and intermediate filaments radiating outward from the nuclear rim. Examples of such antibodies include anti-cytokeratin, anti-vimentin and anti-tropomyosin. Anti-cytokeratin antibodies are often detected in patients with pulmonary conditions such as idiopathic pulmonary fibrosis. Autoantibodies targeting vimentin have been associated with neurofibromatosis type 1 and are considered a potential risk factor for tumour development [16].
2. The speckled cytoplasmic fluorescence pattern is characterized by distinct speckles scattered throughout the cytoplasm. This pattern is frequently associated with autoantibodies directed against cytoplasmic components such as anti-Jo-1 [14].
3. Reticular fluorescence is characterized by anti-mitochondrial antibodies (AMAs). In clinical practice, these antibodies can be found in primary biliary cholangitis (PBC) and in SSc [17,18].
4. Golgi antibodies typically present a distinct perinuclear or crescent-shaped pattern. Proteins targeted by Golgi antibodies include GM130, giantin and TGN46. These have been reported to be potential serologic markers for seronegative RA and many autoimmune diseases [19,20].
5. Rod and ring structures can be identified within the cytoplasm of interphase cells. These patterns were historically associated with patients with hepatitis C virus (HCV) undergoing combination therapy with pegylated interferon-α and ribavirin. The key autoantigen recognized by these antibodies has been identified as nucleotide biosynthetic enzyme inosine monophosphate dehydrogenase (IMPDH). Recent research suggests that these antibodies can be found in individuals from diverse populations and may depend on unidentified internal or external factors [21].

The spindle fibre, intercellular bridge and mitotic chromosomal staining pattern is categorized as mitotic fluorescence [14].

In recent years, work has been carried out to attempt to link the clinical features of the disease with an appropriate serological marker. Data indicates that in healthy and elderly people, a speckled type (nuclear fine speckled or nuclear dense fine speckled) is more commonly observed, followed by a homogeneous type of fluorescence. Therefore, their low titres do not have diagnostic value [22]. In the course of connective tissue diseases, the titre of most antinuclear antibodies does not correlate with the activity of the disease process; therefore, control testing is not recommended for assessing the effectiveness of therapy. So far, the only exceptions to this rule are antibodies directed against ds-DNA, whose presence is associated with renal dysfunction, inflammation of the serous membranes and haematological changes, and correlates with the activity of SLE [3]. Most types of fluorescence are not specific, and some are important in everyday practice. Certain antibodies are specific to certain diseases; for example, anti-dsDNA and anti-Sm are specific to SLE. It is also important to remember that these antibodies have very low sensitivity but very high specificity, making them a valuable tool in the diagnosis of SLE. Anti-Mi2 and anti-Jo1 are specific to dermatomyositis. Antibodies against topoisomerase I (Scl-70) are specific to systemic sclerosis. However, SS-A and SS-B are not specific, as they may be present in people suffering from SLE, sicca (Sjȫgren) syndrome or neonatal lupus erythematosus, but they are common in healthy people. RNP antibodies are popular in mixed connective tissue disease and PM-Scl in overlap syndrome. Most antibodies, however, do not have such close correlations. The characteristics of particular antibodies are described below [3].

### 2.6. Limitations and Pitfalls of ANA Testing

IIF is especially valuable as a first-line screening tool for individuals who are suspected of having a connective tissue disease (CTD), such as systemic lupus erythematosus (SLE), Sjögren’s syndrome, rheumatoid arthritis (RA), mixed connective tissue disease (MCTD), scleroderma (SSc) or polymyositis/dermatomyositis (PM/DM). However, caution should be taken in interpreting positive results, as ANAs may also be present in conditions other than CTD, like Hashimoto’s thyroiditis, Graves’ disease, autoimmune hepatitis, primary autoimmune cholangitis and primary pulmonary hypertension, as well as various infections and malignancies. Moreover, ANA titres may precede the development of any clinical symptoms of autoimmune disease by a number of years. In such cases, treatment should be postponed until the development of warning signs [23]. Also, some drugs, including diuretics, benzodiazepines, bronchodilators and oestrogens, can stimulate ANA formation [24]. It is commonly believed that a higher titre of ANA is associated with a higher positive likelihood ratio to confirm a diagnosis of CTD. Moreover, the presence of a higher titre increases the chance of identifying antibodies in antigen-specific immunoassays. It may be possible to differentiate between healthy people and those with CTD by identifying the ANA pattern. The most common in healthy individuals is the speckled pattern. Healthy adults with positive ANA usually have nuclear dense fine speckled staining; this was found in 33% of healthy people with positive ANA but only in 0.01% of patients with positive ANA with CTD [25]. Furthermore, patients with sunlight-induced skin lesions such as polymorphous light eruption have presented with elevated ANA titres of 1:80 or above, and those with high ANA titres also demonstrate greater persistence of sun-induced skin lesions; the authors also report an increase in ANA titres following UVB-NB and UVA exposure. However, the increase was not correlated with any increased risk of developing CTD [26].

#### 2.6.1. Anti DFS-70 Antibodies

Anti-DFS70 antibodies are named after the dense fine speckled fluorescence pattern they make in IIF assays. These IgG class antibodies bind to the DFS70 protein (70 kDa), commonly referred to as lens epithelium-derived growth factor, which is widely expressed in organisms [27]. The presence of anti-DFS70 antibodies alone at high titres has been reported as a reliable marker for distinguishing between healthy individuals with anti-DFS70 who are ANA positive and those with CTD [28]. Furthermore, the coexistence of anti-DFS70 with other ANA subtypes is a predictive factor of a milder course of CTD. Anti-DFS-70 antibodies have been detected in both healthy people and patients with various inflammatory diseases, like atopic dermatitis, asthma and rheumatoid diseases [29].

#### 2.6.2. Anti-nRNP Antibodies

Anti-nRNP antibodies react with proteins (70 Kd, A, C) associated with U1 RNA to form U1snRNP and are required for the diagnosis of mixed connective tissue disease (MCTD), being present in 95% of patients [30,31]. In contrast, they have been recorded in 30% of patients with SLE. Patients with SLE with anti-nRNP antibodies have lower incidence of severe central nervous system (CNS) involvement or renal disease compared to those with anti-dsDNA antibodies [32,33]. However, recent studies indicate that patients with these antibodies, particularly those with MCTD, can experience severe and potentially life-threatening complications such as hypertension and proliferative vascular lesions [34]. In addition, Hinojosa-Azaola et al. report that patients with SLE presenting with a combination of lupus anticoagulant (LA) and anti-RNP/Sm antibodies demonstrate an increased risk of thrombotic events. Anti-RNP antibodies are also more prevalent in patients with Raynaud’s phenomenon [35].

#### 2.6.3. Anti-Sm Antibodies

Another biomarker of SLE is the presence of anti-Smith (Sm) antibodies, consisting of spliceosomal snRNPs complexed with Sm core proteins [36]. These antibodies are very specific (96–98%) and are useful for confirming diagnosis, even though they are positive in only one-third of patients with SLE (5–40%) [30,37]. Due to their high specificity for SLE, they have been included in the serological diagnostic criteria given by the Systemic Lupus International Collaborating Clinics (SLICC) and American College of Rheumatology and European Alliance of Associations for Rheumatology (ACR/EULAR), formerly the European League Against Rheumatism [38]. Barada et al. report a significant correlation between rising titres of anti-Sm antibodies and clinical exacerbation of both CNS and non-CNS disease in a considerable portion of patients [39]. Increased levels of anti-Sm and anti-NR2 antibodies in cerebrospinal fluid, resulting from blood–brain barrier disruption, are key contributors to the development of an acute confusional state. However, the exact association between anti-Sm antibodies and CNS involvement remains uncertain [40]. A higher prevalence of anti-dsDNA has been noted in patients with serious CNS disease compared to anti-Sm antibodies; hence, it is possible that anti-Sm may be used to identify patients with SLE and milder renal disease and CNS involvement. Janwityanuchit et al. found that patients with anti-Sm antibodies are more likely to suffer from neuropsychiatric manifestations than patients with anti-dsDNA alone [41]. A study conducted on 51 consecutive patients with SLE identified active SLE accompanied by pulmonary, renal and CNS involvement in eight patients with both anti-Sm and anti-dsDNA [42]. A case–control study of patients with SLE revealed that a combination of anti-RNP/Sm antibodies and lupus anticoagulant (LA) could serve as useful parameters in the identification of patients with SLE at risk of thrombosis [43]. The high specificity of anti-Sm antibodies, along with epidemiological evidence, indicates that Epstein–Barr virus infection may trigger the production of anti-Sm antibodies through molecular mimicry [43]. This hypothesis is supported by previous data indicating Epstein–Barr virus infection in the peripheral blood of all 32 of the tested patients with lupus but in only 23 of the 32 matched controls [44].

#### 2.6.4. Anti-dsDNA Antibodies

Anti-DNA antibodies come in two forms: anti-denatured single-stranded DNA (ssDNA) and anti-denatured double-stranded DNA (dsDNA). dsDNA-reactive antibodies mainly recognize epitopes of the double-helix outer framework. The frequency of these antibodies in patients with SLE is estimated in various studies to be between 20 and 90% [45]. Anti-dsDNA antibodies are regarded as a highly specific biomarker for SLE and an important point in the classification of patients with SLE according to the ACR, SLICC and EULAR criteria [46,47]. These antibodies bind to conserved sites of double-stranded DNA and exhibit somatic mutations in their variable regions. They are considered a useful parameter of disease activity [48]. A consistent rise in anti-dsDNA levels corresponds closely with the onset of disease exacerbation [49]. These levels tend to rise during flare-ups of SLE, particularly in lupus nephritis. Various authors have suggested that certain monoclonal anti-dsDNA antibodies can provoke glomerular immune deposits and nephritis in nonautoimmune mice, while others do not [46]. It has been shown that the deposition of immunoglobulins is closely linked to DNA binding. Reducing the affinity of these antibodies for DNA could potentially eradicate glomerular deposition and nephritis [50,51]. Anti-dsDNA antibodies have been found to have pathogenic effects in lupus nephritis, dermatitis and certain forms of cerebral lupus. However, the determination of these pathogenic pathways, whether through cross-reaction with non-DNA structures or via homologous recognition of dsDNA exposed in different parts of DNA remains uncertain [47]. As these antibodies are associated with other autoimmune syndromes, as well as with bacterial [48], viral [49] and parasitic infections [48] and cancers, SLE should not be immediately suspected in every patient with these antibodies. In these clinical situations, anti-dsDNA is present in low to moderate titres. Importantly, these sets of anti-dsDNA antibodies are specific to unique DNA structures [52,53,54]. The estimated frequency of anti-dsDNA is highly dependent on the methods used and the disease activity in patients with SLE included in the studies. Practically, the presence of anti-dsDNA can be determined through various methods, including enzyme-linked immunosorbent assay (ELISA), radioimmunoassay (RIA), the crithidia luciliae immunofluorescence test (CLIFT), Addressable Laser Bead Immunoassay (ALBIA) and Fluorescent Enzyme Immunoassay (FEIA). Despite there being a benchmark for measuring antibodies, there are still challenges associated with that measurement. The ELISA test is popular but preferentially detects low-avidity anti-dsDNA antibodies that are not as specific for SLE. Therefore, it is advised that any positive anti-dsDNA ELISA findings be verified using a second testing method that specifically detects high-avidity anti-dsDNA, such as the Farr radioimmunoassay or CLIFT [55]. CLIFT analysis offers greater clinical specificity than any solid phase-based assay. The test uses flagellates of the species Crithidia luciliae to detect dsDNA; the species has mitochondria containing dsDNA in which there are no other nuclear antigens. Antibodies reacting with this dsDNA are directed exclusively against double-stranded DNA. Therefore, many researchers propose a combined detection strategy based on IIF, ELISA and CLIFT as a rational diagnostic approach [56]. A study examined the presence of anti-dsDNA antibodies in 1073 people with recent onset of rheumatic symptoms using two different CLIFT kits. Among these, a diagnosis of SLE was initially made in 65 patients, of whom 24 (37%) were CLIFT positive. Of the patients who were ANA negative, 16 (5.5%) were CLIFT positive. After approximately five years, of the 36 patients who were CLIFT positive and not diagnosed with SLE at study entry, only one developed SLE during the follow-up period. These findings suggest that patients without SLE who are CLIFT positive have little risk of developing SLE within five years [57].

#### 2.6.5. Anti-Ro/SS-A and Anti-La/SS-B Antibodies

Although anti-Ro/SS-A antibodies are among the most frequently detected autoantibodies against extractable nuclear antigens and are the most prevalent autoantibodies among many autoimmune diseases, their pathological role remains controversial. It has, however, been demonstrated that their presence could precede the first symptoms of disease by many years. They are frequently associated with anti-La/SS-B antibodies but can be detected alone in rare cases. They were first described in 1961 by Anderson et al. in a patient with SS [58] and then later in a patient with SLE, when they were named anti-Ro antibodies [59]. Later, antibodies to another soluble cytoplasmic RNA protein were isolated and named anti-La antibodies. As the described antibodies were found to be antigenically identical to SS-A and SS-B, they are referred to by the double names anti-Ro/SS-A and anti-La/SS-B [60]. Ro/SS-A and La/SS-B antigens share common structural characteristics with other cellular antigens (Sm, U 1RNP)—they are associated with several small RNAs, approximately 100-nucleotides long, and form Ro-ribonucleoprotein particles [61]. Later, Ro antigens were found to consist of two different proteins, Ro60 and/or Ro52. Anti-Ro52 antibodies can exist independently without the presence of anti-Ro60 [62]. Many patients with autoimmune myositis present with anti-Ro-52 antibodies, particularly monospecific ones. These antibodies are strongly linked with myositis-specific autoantibodies targeting aminoacyl-tRNA synthetases (AATS) [63,64]. A large multicentre cohort study found anti-Ro/SSA and/or anti-La/SSB antibodies to be associated with mild manifestations of systemic lupus erythematosus (SLE), with a particular emphasis on cutaneous and musculoskeletal involvement. These antibodies are also very specific markers for Sjögren syndrome (SSA) [65]. While positive ANA is detected in up to 70% of Sjögren syndrome cases, anti-Ro/SS-A and anti-La/SS-B antibodies are present in smaller proportions. Approximately 48% of patients with Sjögren syndrome test positive for anti-Ro/SS-A antibodies, with 39% showing positivity at the time of diagnosis. The presence or absence of these antibodies is associated with distinct clinical phenotypes; for instance, patients who are ANA positive and negative for Ro/SS-A and La/SS-B antibodies tend to exhibit a lower frequency of peripheral neuropathy [66]. In turn, patients with Ro and La are significantly more likely to demonstrate earlier disease onset, longer disease duration, parotid or major salivary gland enlargement and intensive lymphocytic infiltrations of the minor salivary glands [67,68]. Moreover, these antibodies contribute to lymphadenopathy, splenomegaly and vasculitis. Positive antibodies to Ro/SS-A may also be found in patients with subacute cutaneous lupus erythematosus. As anti-Ro/SS-A antibodies can be transferred from a pregnant woman to the foetus, potentially leading to neonatal manifestations such as congenital heart block, it is recommended that pregnant women with SLE and/or Sjögren syndrome be screened for these antibodies to assess the risk to the foetus [69,70]. Although anti-Ro/SS-A antibodies are detected mainly in patients with subacute systemic lupus erythematosus (SCLE), SS and NLE, they are also found in 3–11% of patients with systemic sclerosis (SSc); they are also associated with sicca symptoms, severe pulmonary involvement, idiopathic inflammatory myopathy, rheumatoid arthritis, primary biliary cholangitis (PBC) and an autoimmune liver disease. Anti-Ro52 antibodies are mostly associated with myositis, SSc and PBC [71]. Anti-Ro/SS-A antibodies are also minimally expressed in HEp-2 cells. As such, in cases where SS, SCLE or NLE are suspected, the diagnostician should consider using Hep-2000 as a substrate or using ELISA, even when ANA findings are negative [71].

#### 2.6.6. Anti-Scl-70

Anti-Scl-70 is a subtype of ANAs that has been included in the routine diagnosis of SSc for about three decades [72]. They bind to topoisomerase I, a nuclear protein with a molecular weight of 70–100 kD, which is needed for efficient DNA replication and transcription. Topoisomerase I enables the cutting of a single DNA strand, preventing super-strand formation and conditioning the relaxation of the DNA molecule [73]. Anti-Scl-70 antibodies exhibit a prominent fine speckled pattern in IIF [72]. The antibodies are highly specific for SSc and have been noted in approximately 60% of patients with SSc. Furthermore, anti-Scl 70 is associated with the development of a diffuse form of SSc, characterized by widespread skin involvement by sclerosis and a severe course. Patients with anti-Scl-70 antibodies have been shown to have an increased risk of certain effects: forming ischaemic digital ulcerations at the early stage of the disease, flexion contractures of the palms leading to deformities and reduced mobility and lung fibrosis and even neoplastic processes of various origins [74,75]. The presence of anti-Scl-70 antibodies is also a proven risk factor for the development of interstitial lung disease (ILD), the most severe organ complication among patients with SSc, with the highest mortality rate. Compared to anti-centromere antibodies (ACAs), the second most frequently detected antibodies in the plasma of patients with SSc, anti-Scl-70 antibodies show a stronger correlation with the incidence of conditions such as synovitis, muscle involvement, heart block and respiratory disorders such as ILD and restrictive lung disease [76,77]. Normally, anti-Scl-70 antibodies are classified as IgG in laboratory diagnostics. Cavazzana I et al., in their study, found that anti-Scl70 correlates with disease activity and indicates increased risk for pulmonary hypertension and nephritis [78]. Additionally, anti-topo I is associated with diffuse skin involvement and pulmonary fibrosis [79]. However, it should be noted that anti-Scl-70 could be present in other CTDs, including SLE, without clinical symptoms of SSc; in such cases, they are accompanied by other antibodies. When present, they correlate with disease activity and indicate increased risk of pulmonary hypertension and nephritis [78].

#### 2.6.7. Anti-PM/Scl Antibodies

Anti-PM/Scl antibodies are directed against several antigenic components of the PM/Scl multi-subunit complex, i.e., the human exosome; these include the two main proteins PM/Scl-75 (75 kD) and PM/Scl-100 (100 kD). The human exosome is responsible for the processing of different RNA subtypes and their degradation. Anti-PM-Scl antibodies are characterized by a homogenous nucleolar type of fluorescence (AC-8) in IIF [72,80]. They are mostly detected in the serum of patients with SSc, PM and DM, as well as SSc/PM overlap syndrome [80]. A meta-analysis of studies found that anti-PM/Scl antibodies are present in 31% of patients with PM/Scl overlap syndrome, in 11% of those with DM, in 8% with PM and in 2% with SSc [63,81]. Of note, patients who are anti-PM/Scl positive are more likely to present symptoms such as ILD, muscle involvement with muscle weakness and myalgia, arthritis, Raynaud’s phenomenon, typical symptoms of DM (Gottron’s sign, heliotrope sign and, less frequently, mechanic’s hands), sclerodactyly, fevers unrelated to infection and dysphagia [82,83]. It should be emphasized that anti-PM/Scl positivity is closely correlated with a high risk of developing ILD in the course of the underlying disease. A study on a Chinese population found that as many as 86.7% of patients with detected anti-PM/Scl were diagnosed with ILD and 10% were diagnosed with mild pulmonary arterial hypertension [83]. However, it has been proven that ILD develops slowly in patients with SSc, resulting in a slightly lower mortality rate in the first 10 years of the disease [72]. No significant differences in the frequency of individual symptoms were found between patients with anti-PM/Scl-75 and those with anti-PM/Scl-100. However, the relationship between the occurrence of anti-PM/Scl antibodies and the presence of internal tumours remains unclear [72]. Differentiating between anti-PM/Scl-75 and anti-PM/Scl-100 antibodies is useful because they may indicate different clinical manifestations. One study showed that anti-PM/Scl-75 antibodies were detected more frequently in younger and more active patients with joint contractures, whereas nti-PM/Scl-100 antibodies were associated with creatine kinase elevation and lower incidence of gastrointestinal involvements [84].

#### 2.6.8. Anti-Ribosomal P Protein

Anti-ribosomal P protein antibodies (anti-Rib-P) have been found to act as markers of SLE with low sensitivity but high specificity; as such, their presence may be used to improve the accuracy of diagnosis. They occur with a frequency of only 10–40% among patients with SLE and vary somewhat according to population type and detection method. Anti-Rib-P is extremely rare among the healthy population and also among patients diagnosed with autoimmune diseases other than lupus [85]. However, these antibodies could be detected in 10% of patients with autoimmune hepatitis without SLE, and their presence seems to be connected with worse prognosis [86]. The occurrence of anti-Rib-P antibodies differs significantly across ethnic groups, with a notably higher prevalence in Asian patients with SLE (36%) than in Caucasian and African American patients (15%) [87,88]. Anti-Rib-P antibodies target a common epitope for three ribosomal phosphoproteins, viz., P0 (38 kD, P1 (19 kD) and P2 (17 kD). In the 60S subunit of the ribosome, these molecules form a pentameric complex consisting of one P0 monomer and two P1/P2 dimers. This complex binds to the rRNA of the ribosome and is involved in the elongation of the amino acid chain during the translation process [89]. It has been suggested that anti-Rib-P antibodies may predict the occurrence of specific manifestations in the course of SLE, such as neuropsychiatric, renal, articular, hepatic, skin and haematological abnormalities, or juvenile SLE. However, studies have revealed some inconsistencies. Zhen-rui et al. report a positive correlation between the presence of anti-Rib-P antibodies directed against various phosphoproteins and disease activity, as well as the occurrence of skin lesions; however, they do not report any such relationship for oral mucosal involvement, serositis, neuropsychiatric manifestations or renal and haematological disorders [89]. Interestingly, of the 19 patients with SLE with negative anti-dsDNA and anti-Sm antibodies, eight individuals (42%) had positive anti-Rib-P antibodies. Therefore, anti-Rib-P antibodies, alongside anti-dsDNA and anti-Sm, may be an adjunctive marker in the diagnosis of SLE [89]. Patients with SLE presenting with anti-dsDNA and anti-Rib-P antibodies have been found to experience milder kidney dysfunction than those with anti-dsDNA alone. This may indicate that anti-Rib-P antibodies possess nephroprotective properties in such cases [89].

#### 2.6.9. Anti-Jo-1 Antibodies

The most common type of anti-synthetase antibody is believed to be anti-Jo-1, which is directed against the cytoplasmic protein histidyl-tRNA synthetase. This enzyme is thought to enable the binding of histidine to tRNA during protein translation [90]. Anti-Jo-1 antibodies are typically found in patients with idiopathic inflammatory myopathies (IIMs) with a frequency of 15–30%. The IIM group of diseases includes various connective tissue diseases, such as PM, DM, inclusion body myositis, myositis associated with malignancy, immune-mediated necrotizing myopathy and antisynthetase syndrome (ASS). Among these entities, anti-Jo-1 is most prevalent and fairly specific in PM/DM [90]. Histidyl-tRNA synthetase, the target autoantigen, is overexpressed in lung and muscle tissue [91]. Therefore, the presence of these antibodies is believed to indicate poorer prognosis in the development of IIMs, but also ILD [92]. The presence of anti-Jo-1 correlates with the typical clinical phenotype, consisting of idiopathic ILD, inflammatory muscle disease and non-erosive arthritis, making up the classic ASS triad; in addition, it is associated with Raynaud’s phenomenon, “mechanic’s hands”, fever and frequent relapses [92]. Interestingly, among patients with ASS, individuals who were anti-Jo-1 positive were less likely to present with pulmonary arterial hypertension than those who were anti-Jo-1 negative [93]. Approximately 5–8% of patients with ASS may develop an overlap syndrome with another connective tissue disease such as SLE, RA, SSc or SS [92]. Some reports suggest a positive correlation between the titre of anti-Jo-1 antibodies and disease activity, indicating that these antibodies may be a potential marker of IIM activity. However, further studies are required to confirm this relationship [94,95].

#### 2.6.10. Anti-Centromere B Protein

Anti-centromere antibodies (ACAs) are common antibodies in the course of various autoimmune diseases. They are most characteristic of limited cutaneous SSc (lcSSc), in which they occur with a frequency of up to 80%; in contrast, they are found in only 10% of patients with diffuse SSc. ACAs can also be detected in SS, primary biliary cholangitis, SLE, RA, Raynaud syndrome or overlap syndromes [96,97]. In IIF, they reveal a discrete speckled pattern [98]. The target antigens for ACAs are the centromeric proteins involved in the process of cell division; these form a centromeric region, the so-called kinetochore, which is a chromosome fragment involved in chromosome segregation during mitosis. The main centromere-specific proteins include centromeric proteins A (CENPA), B (CENPB) and C (CENPC), with molecular masses of 17 kD, 80 kD and 140 kD, respectively [96,97]. As anti-CENPB proteins are widely prevalent in lSSc, they have been associated with the clinical features formerly referred to as CREST syndrome, i.e., calcinosis, Raynaud’s phenomenon, oesophageal dysmotility, sclerodactyly and telangiectasia [99].

There are some reports of an association between the presence of anti-CENPB and cancer. Atalay et al. indicate a four-fold higher prevalence of anti-CENPB antibodies among patients with breast cancer after prior exclusion of autoimmune disease compared to a control group with benign conditions (33% vs. 8%) [100]. It is therefore presumed that the determination of anti-CENPB antibodies in women with premalignant breast lesions, such as atypical epithelial hyperplasia or lobular carcinoma in situ, can be used as an adjunctive test for the early detection of breast cancer [100]. Interestingly, the results of a study conducted on the same cohort five years later by the same author indicated a significant positive correlation between the presence of anti-CENPB antibodies and disease-free and overall survival among patients with breast cancer [101]. It has also been found that anti-CENPA and anti-CENPB autoantibodies are overproduced in patients with colorectal cancers due to increased transcription of CENP-A and CENP-B genes [102].

#### 2.6.11. Anti-Nucleosome Antibodies—ANuAs

The nucleosome is a core octameric unit of chromatin wrapped in helical DNA; it comprises two copies each of histones H2A, H2B, H3 and H4. While ANuAs are detected in various connective tissue diseases, including SLE, scleroderma and mixed connective tissue disease, they demonstrate their highest sensitivity and specificity, higher even than anti-dsDNA, towards SLE. A study on a group of 90 patients found that aNuAs demonstrated 52% sensitivity and 98% specificity for detecting SLE compared to 37% and 97% for anti-dsDNA [103]. Nucleosomes are exposed on the cell surface during apoptosis and become a key immunogen in SLE [104]. They enhance interleukin-6 production, inhibit phagocytosis and regulate the function of glomerular mesangium cells [105]. Despite this, anti-nucleosome antibodies are not included in the 2019 EULAR/ACR classification criteria for SLE [10]. ANuAs appear in 70–80% of the SLE population, mainly in young untreated patients. They are predominantly found in the early phase of SLE, often in drug-induced lupus erythematosus, usually preceding the production of anti-dsDNA and anti-histone antibodies; they are believed to present a more significant positive correlation with SLE activity than anti-dsDNA [106]. AnuAs and anti-dsDNA often coexist in patients with SLE and are indicative of high disease activity and increased risk of lupus nephritis [104]. They bind components of the glomerular basement membrane and participate in the formation of immune complexes deposited in the kidneys. They also mediate the binding of other autoantibodies to the basement membrane [107]. Chromatin units contained in immune deposits can be both a target and an inducer of anti-dsDNA and aNuA production [103]. However, to a lesser extent, aNuAs have been associated with haematological disorders, arthritis, pleuritis and skin involvement [107,108,109]. ANuAs are also present in the serum of patients with primary antiphospholipid syndrome, where they may act as a predictor of future SLE development [106]. Therefore, the detection of aNuAs in the case of an AC-1 positive pattern with negative anti-dsDNA antibodies may be a relatively simple and quick complementary test for the differential diagnosis of SLE. ANuAs can also be used for assessing the effectiveness of SLE management [100].

#### 2.6.12. Anti-Histone Antibodies (AHAs)

Another clinically relevant subtype of antinuclear antibodies are anti-histone antibodies (AHAs), which are directed against the histone octamer subunits of the nucleosome. AHAs bind to individual free histones, sometimes without showing cross-reactivity, as well as to DNA–protein complexes to form transcriptionally inactive chromatin [110]. The target antigens include the core histones (H2A, H2B, H3, H4) and the linker histone H1. The strongest antigen–AHA interaction is for histones H1, H2A and H2B. Histones may be overexpressed as a result of apoptotic blebs or neutrophil extracellular trap (NET) activity, which, together with post-translational modifications, can affect their immunogenicity [111]. AHAs are among the so-called damage-associated molecular patterns (DAMPs), which activate non-specific immunity mechanisms and repair damaged tissues in stress situations. DAMPs can also promote inflammation in autoimmune diseases, such as RA, SLE, MCTD, Sjögren’s syndrome and inflammatory myositis, but also in neurodegenerative diseases and cancer. AHAs are most commonly detected as IgG class, with IIF usually revealing an homogenous staining pattern [112]. The presence of AHA is typically attributed to SLE and DILE. In DILE, AHAs are detected more frequently than in classic SLE. AHAs have been found to demonstrate 67% sensitivity and 95% specificity towards DILE [113]. In patients with SLE, AHAs occur at a frequency of approximately 50%, with a sensitivity of 55–92% and specificity of 69–82%. The most characteristic AHAs of SLE are directed against H1, H3 and H4, and their presence can support a diagnosis of SLE where diagnosis is complicated. However, it should be noted that AHAs show lower sensitivity than aNUAs and lower specificity than anti-dsDNA in the course of SLE [112]. AHAs are associated with renal involvement in SLE and the occurrence of lupus nephritis. There are reports of a positive correlation between AHA levels and LN exacerbation, but their presence does not appear to affect overall disease activity in SLE [114,115,116]. Previous studies suggest a possible link between AHAs and oral ulcers, neurodegeneration, lymphopenia and fatigue. However, no association between AHAs and skin involvement or arthritis has been proven [112]. Interestingly, injections of a histone deacetylase inhibitor were found to reduce LN activity in a murine SLE model. This demonstrates the potential for targeted post-translational modifications of proteins as a form of adjunctive treatment for SLE [117].

#### 2.6.13. Antibodies Directed Against Proliferating Cell Nuclear Antigen (PCNA)

PCNA is detected in different systemic autoimmune rheumatic diseases, with the highest frequency in SLE. PCNA has low sensitivity but high specificity towards SLE [117]. The target nuclear protein with a molecular weight of 34 kD is a cofactor of DNA polymerase delta and is part of a multiprotein complex that regulates the cell cycle and is involved in DNA strand synthesis [118]. PCNA reveals a characteristic nuclear speckled pattern in IIF [119]. Interestingly, the level of PCNA expression reflects the level of cellular proliferation in both healthy and tumour tissues, and it may be the case that PCNA is involved in the process of carcinogenesis [120]. In SLE, elevated PCNA titres have been reported to correlate with a severe disease course with renal and central nervous system involvement as well as haematological abnormalities [121,122]. However, reports on the clinical significance of PCNA antibodies are conflicting, with some confirming an association between the presence of high levels of PCNA and the occurrence of seizures and renal and joint involvement. Some studies do not note any increased prevalence of PCNA antibodies in SLE [114,123,124]. As PCNA levels reflect the proliferative potential of cells, they are an important prognostic factor for cancer. Elevated PCNA titres are found in breast, duodenal and lung cancer, as well as in malignant lesions of the oral and nasal epithelium. Furthermore, PCNA levels positively correlate with tumour stage in breast cancer, based on the TNM classification [125,126]. Higher expression of proliferating cell nuclear antigen was observed in non-small cell lung cancer (NSCLC) than in adjacent tissues, supporting the involvement of PCNA in the pathogenesis of NSCLC. Furthermore, patients with higher expression of proliferating cell nuclear antigen presented a shorter median survival time than those with low expression. Elevated titres of PCNA have also been noted in people with hepatitis B and C virus.

#### 2.6.14. Anti-Mitochondrial Antibodies (AMAM2)

Anti-mitochondrial antibodies (AMAs) are directed against a wide spectrum of mitochondrial antigens, including proteins of the inner and outer mitochondrial membrane. Nine subtypes of AMAs have been distinguished, viz., AMA M1-M9 [127]. The AMAM2 subtype targets lipoic acid-containing immunodominant epitopes, including the E2 subunits of 2-oxo acid dehydrogenase complexes (PDC-E2) [128]. IIF detection of AMA M2 in rat kidney, stomach and liver tissue, or HEp-2 cell lines as substrates, reveals uniform granular cytoplasmic staining [129]. AMA M2 shows the highest specificity against primary biliary cholangitis and remains its key serological marker. Their elevated titres have also been shown in SLE, SSc, SS, inflammatory myopathies, autoimmune hepatitis type 1, chronic viral hepatitis, infections and cardiovascular disorders [124]. Up to 95% of patients with PBC present with AMA M2. Together with elevated alkaline phosphatase levels and histopathological features of cholangitis, a positive AMA M2 antibody result is one of the three main diagnostic criteria for PBC. Furthermore, the presence of AMA M2 often precedes the other symptoms of the disease [124,130]. The few patients with PBC who are AMA M2 negative may require a liver biopsy in the course of diagnosis. AMA M2 may also facilitate the rapid diagnosis of PBC, thus avoiding serious complications [131]; importantly, it should be noted that AMA M2 has no prognostic value in PBC, as its levels do not correlate with disease activity or treatment efficacy [130]. Despite being detected in patients with SLE with a frequency of about 10%, AMA M2 demonstrates no diagnostic value in the disease, as no clear associations have been established between the presence of AMA M2 and the clinical picture in SLE. To date, it has not been clarified whether the presence of AMA M2 antibodies in SLE increases the risk of developing PBC in the future. Interestingly, patients with SLE who are AMA M2 positive usually have liver enzyme values within the normal range or mild cholestasis. The risk of developing PBC in this group is less than 1%. Therefore, confirmation of this relationship requires further research [130]. AMA M2 antibodies are also involved in the pathogenesis of IIM. In this group, AMA M2 occurs with a frequency of 2.5% to 19.5%. Detection of AMA M2 is then associated with certain clinical features, such as a chronic disease course, less severe muscle weakness, severe cardiac and pulmonary involvement, including cardiomyopathy and respiratory failure, as well as weight loss. Nagai et al. report that various forms of immunotherapy can alleviate muscle weakness and improve respiratory function [132]. All of the antibodies are summarized in Table 2. 

## 3. Conclusions

In conclusion, ANAs are used worldwide as serological biomarkers for screening and have played a part in the research, diagnosis, classification and phenotyping of systemic and organ-specific autoimmune diseases. ANAs are a sensitive, but mostly not specific, marker of CTD. Only a few correlate with disease activity in SLE; of these, anti-dsDNA has been proven to be effective, and aNuAs, AHAs and PCNA have been found to offer promise in clinical trials. ANAs may also be associated with specific clinical features, such as anti-Sm, associated with central nervous system involvement in SLE, anti-dsDNA with lupus nephritis, anti-Scl 70 with a diffuse form of SSc or anti-Jo-1 with ILD. The gold standard for detection is IIF with HEp-2 cells as a substrate and with dozens of autoantibodies directed against numerous intracellular antigens; the resulting data can provide information on serum levels and possible antigenic targets. Laboratory tests are only useful if requested in the appropriate clinical context.

## Figures and Tables

**Table 1:** Main fluorescence patterns.

	Fluorescence Pattern	Associated Specificities
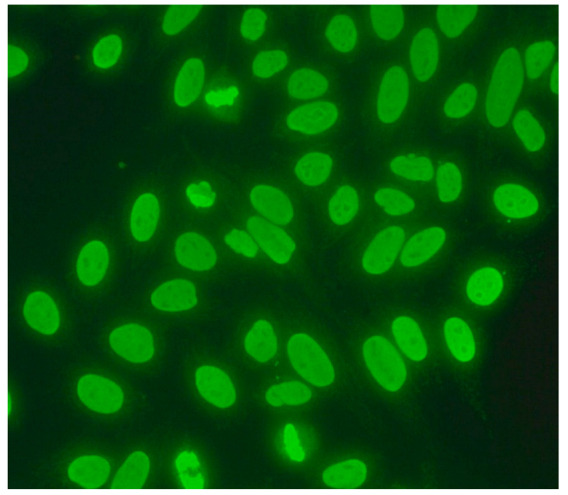 1:600	Homogeneous pattern (AC-1) Homogeneously dispersed nuclear fluorescence can be distinguished in the preparation by an intensely and uniformly stained chromatin mass in mitotic cells.	Anti-dsDNA ^1^ Anti-histone Anti-nucleosome
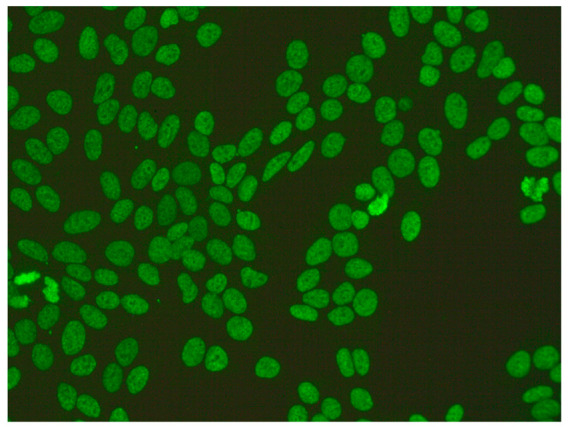 1:600	Nuclear dense fine speckled pattern (AC-2) A grainy pattern evenly dispersed in the nucleus, characterized by different sizes, fluorescence intensity and speckled distribution. The metaphase plate shows a strongly mottled pattern with thick spots.	Anti-DFS-70 ^2^
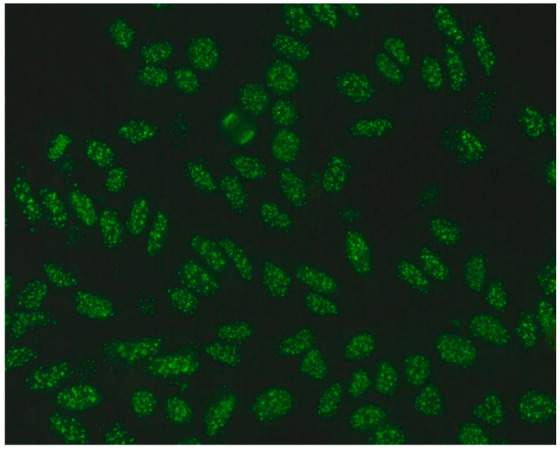 1:600	Centromere pattern (AC-3) Discrete coarse speckles scattered in interphase cells and aligned at the chromatin mass on mitotic cells.	Anti-Centromere A protein Anti-Centromere B protein
1:600 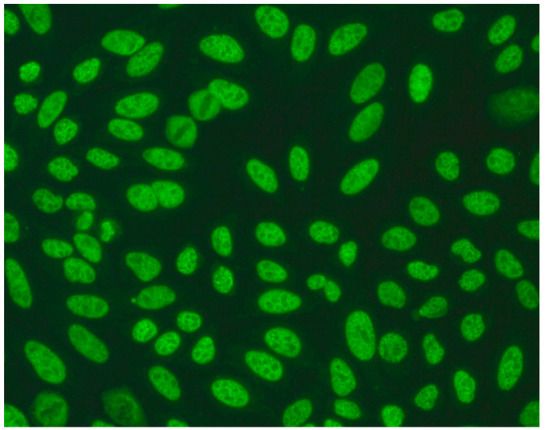	Nuclear fine speckled (AC-4) Fine-grained fluorescence dispersed evenly throughout the nucleus of the cell. In addition, an unstained chromatin mass of mitotic cells can be observed in the preparation.	Anti-SS-A/Ro ^3^ Anti-SS-B/La ^4^ Anti-Mi-2 ^5^ Anti-TIF1γ ^6^ Anti-TIF1β ^7^ Anti-Ku ^8^
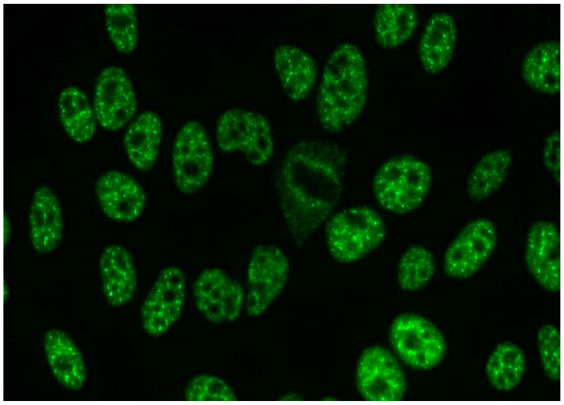 1:600	Nuclear large/coarse speckled pattern (AC-5) Coarse speckles across all nucleoplasm. The nucleoli may be stained or not stained. The chromatin mass in mitotic cells (metaphase, anaphase and telophase) is not stained.	Anti-Sm ^9^ Anti-U1 RNP _10_ Anti-RNA polymerase III
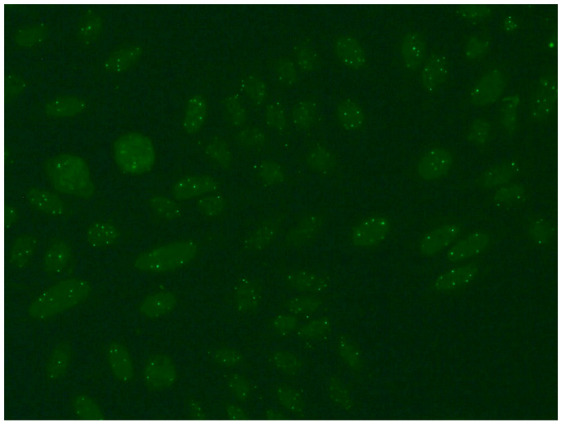 1:600	Multiple nuclear dot pattern (AC-6) Countable discrete nuclear speckles	Anti-Sp-100 ^11^ Anti-PML antibodies ^12^ Anti-MJ/NXP-2 antibodies ^13^
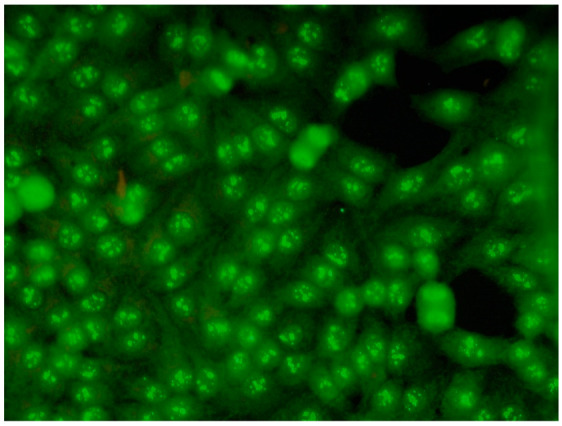 1:600	Nucleolar pattern Characteristic staining of nucleoli in the cell, which, depending on the antigens in their structures, show differentiated fluorescence. We can distinguish three types of nucleolus fluorescence—homogeneous (AC-8), clumpy (AC-9), punctate (AC-10). The homogeneous type is characterized by diffuse, intense coloration of the nucleoli. The clumpy type shows irregular fluorescence in terms of intensity and uniformity. In the punctate nucleolar, there are distinct, densely distributed grains	Anti PM/Scl-75 ^14^ Anti PM/Scl-100 ^15^ Anti Th/To ^16^ Anti mU3-snoRNP ^17^ RNA polymerase I, Anti hUBF/NOR-90 ^18^
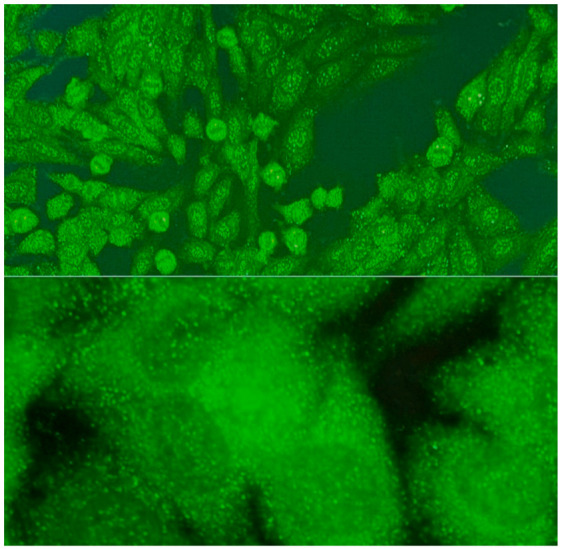 1:600	Speckled cytoplasmic pattern (AC-18) Characterized by distinct speckles scattered throughout the cytoplasm. This pattern is frequently associated with autoantibodies directed against cytoplasmic components.	Anti-Jo-1 ^19^ Anti-SRP ^20^
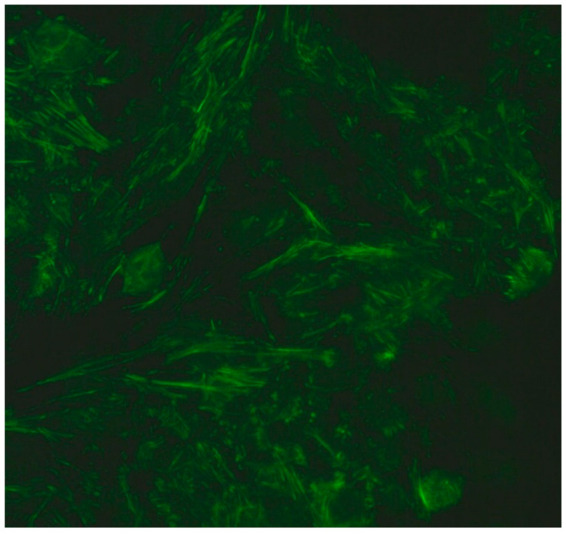 1:600	Cytoplasmic fibrillar linear pattern (AC-15) Characterized by decorated cytoskeletal filaments, sometimes with small, discontinuous granular deposits. Typical staining shows striated actin cables spanning the long axis of the cells.	Anti-actin Anti-non-muscle myosin
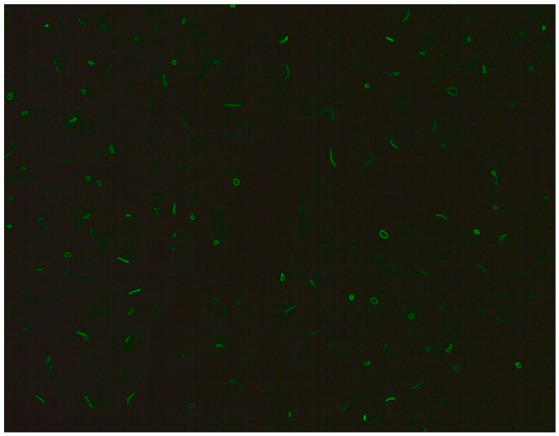 1:600	Rod and ring pattern (AC-23) Visible intracellular polymer structures in the form of rings and rods are located within the cytoplasm of cells.	anti-IMPDH2 ^21^
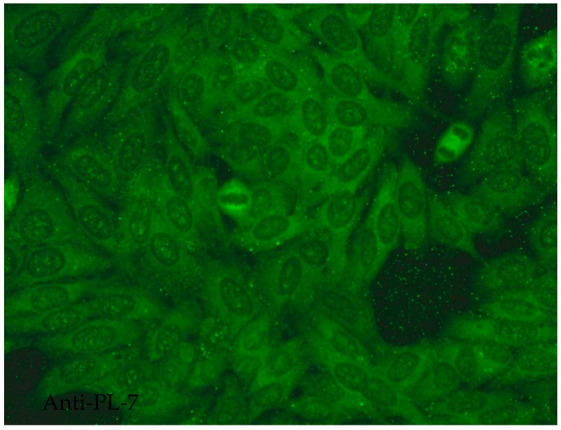 1:600 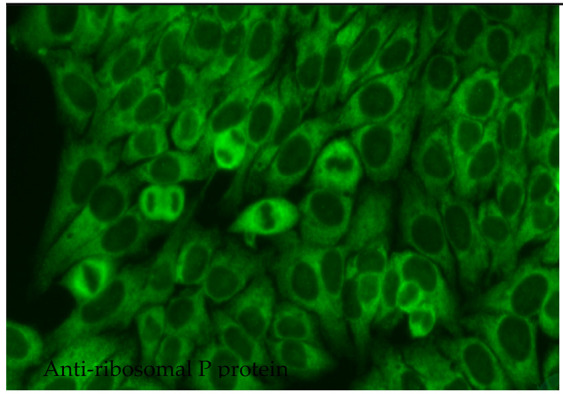 1:600	Cytoplasmic dense fine speckled pattern (AC-19) The pattern appears cloudy, almost homogeneous throughout the cytoplasm.	Anti-PL-7 ^22^ Anti-PL-12 ^23^ Anti-ribosomal p protein
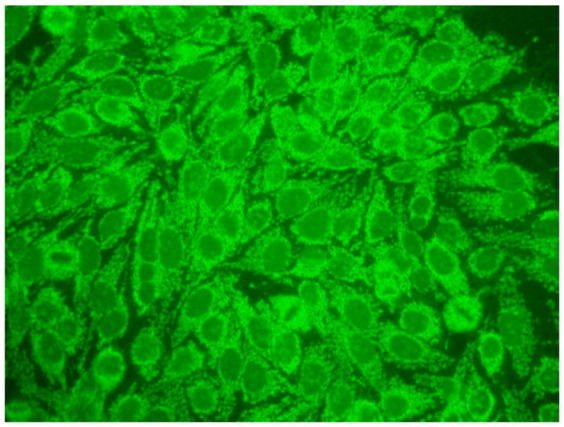 1:600	Cytoplasmic reticular/AMA pattern (AC-21) Coarse granular filamentous staining extending throughout the cytoplasm.	Anti-mitochondrial

^1^ Autoantibodies against double-stranded DNA antibody. ^2^ Autoantibodies against dense fine speckled 70 antibodies. ^3^ Autoantibodies against Sjogren’s syndrome antigen A (Ro). ^4^ Autoantibodies against Sjogren’s syndrome antigen B (La). ^5^ Autoantibodies against myositis autoantigen Mi-2. ^6^ Autoantibodies against transcription intermediary factor 1-gamma. ^7^ Autoantibodies against transcription intermediary factor 1-beta. ^8^ Autoantibodies against Ku autoantigen. ^9^ Autoantibodies against Smith. ^10^ Autoantibodies against U1 ribonucleoprotein antibodies. ^11^ Autoantibodies against Sp100 protein. ^12^ Autoantibodies targeting the promyelocytic leukaemia protein. ^13^ Autoantibodies against NXP-2 (nuclear matrix protein), also known as anti-MJ antibodies. ^14^ Autoantibodies against the 75 kDa protein in the polymyositis/scleroderma (PM/Scl) complex. ^15^ Autoantibodies against the 100 kDa protein in the polymyositis/scleroderma (PM/Scl) complex. ^16^ Autoantibodies directed against Th and To ribonucleoprotein antigens. ^17^ Autoantibodies against the U3 small nucleolar RNP complex. ^18^ Autoantibodies against human upstream binding factor, nucleolar organizer region-90 kDa protein. ^19^ Autoantibodies against histidyl-tRNA synthetase. ^20^ Autoantibodies against the signal recognition particle. ^21^ Autoantibodies against inosine monophosphate dehydrogenase type 2. ^22^ Autoantibodies against threonyl-tRNA synthetase. ^23^ Autoantibodies against alanyl-tRNA synthetase.

**Table 2:** Summary table of autoantibodies: clinical importance, sensitivity and specificity.

Autoantibody	Associated Diseases	Clinical Importance	Sensitivity	Specificity
Anti-dsDNA	SLE (mainly lupus nephritis)	Disease activity marker; renal involvement; included in ACR/EULAR criteria	20–90% (method-dependent)	High (often >95%)
Anti-Sm	SLE	Highly specific diagnostic marker; associated with CNS involvement	5–40%	96–98%
Anti-nRNP	MCTD, SLE	Key marker for MCTD (95%); milder CNS/renal involvement in SLE	30% in SLE; 95% in MCTD	Moderate
Anti-SSA/Ro	SS, SCLE, NLE, SLE, SSc	Common in SS and SCLE; risk of neonatal lupus and congenital heart block	~48% in SS	Moderate
Anti-SSB/La	SS, SLE	Often co-occurs with SSA/Ro; early disease marker	Lower than SSA	Moderate
Anti-Scl-70	Diffuse SSc	Diffuse cutaneous SSc; ILD risk; associated with worse prognosis	~60% in SSc	High (~95%)
Anti-centromere B protein	Limited SSc (CREST)	Associated with CREST syndrome; slow disease progression	~80% in lcSSc	High
Anti-PM/Scl	PM/Scl overlap, SSc, DM	ILD, muscle involvement, overlap syndromes	2–31% (varies by disease)	High
Anti-Jo-1	ASS, PM/DM	ILD and myositis marker; poor prognosis; classic antisynthetase syndrome	15–30% in IIM	High
Anti-Ribosomal P protein	SLE, autoimmune hepatitis	CNS involvement, juvenile SLE; adjunct marker when dsDNA/Sm negative	10–40% in SLE	High (~95%)
Anti-PCNA	SLE, cancers	Controversial; possibly associated with severe SLE and malignancy	Low	High
Anti-histone	DILE, SLE	Key marker in DILE; also seen in SLE; renal involvement	55–92% in SLE	69–95%
Anti-nucleosome (ANuA)	SLE, DILE, MCTD	Early SLE marker; better correlation with disease activity than dsDNA; nephritis predictor	52–80% in SLE	97–98%
Anti-DFS70	Healthy individuals, dermatitis, mild CTD	Exclusion marker for systemic autoimmunity when isolated	Detected in many healthy individuals	Low for CTD
AMA-M2	Primary biliary cholangitis, SLE, IIM	Specific marker for PBC; rare in SLE without liver symptoms	~95% in PBC	Very High

## Abbreviations

The following abbreviations are used in this manuscript:

ANAs	Antinuclear antibodies
SLE	Systemic lupus erythematosus
IIF	Indirect immunofluorescence assay
ELISA	Enzyme-linked immunosorbent assay
ICAP	The International Consensus on ANA Patterns
CTDs	Connective tissue diseases
DILE	Drug-induced lupus erythematosus
PBC	Primary biliary cholangitis
PM/DM	Polymyositis/dermatomyositis
SSc	Systemic scleroderma
RA	Rheumatoid arthritis
RIA	Radioimmunoassay
CLIFT	Crithidia luciliae immunofluorescence test
ASS	Antisynthetase syndrome
ACAs	Anti-centromere antibodies
AHAs	Anti-histone antibodies
DAMPs	Damage-associated molecular patterns
PCNA	Antibodies directed against proliferating cell nuclear antigen
AMAs	Anti-mitochondrial antibodies
